# Intravenous multipotent adult progenitor cell treatment decreases inflammation leading to functional recovery following spinal cord injury

**DOI:** 10.1038/srep16795

**Published:** 2015-11-19

**Authors:** Marc A. DePaul, Marc Palmer, Bradley T. Lang, Rochelle Cutrone, Amanda P. Tran, Kathryn M. Madalena, Annelies Bogaerts, Jason A. Hamilton, Robert J. Deans, Robert W. Mays, Sarah A. Busch, Jerry Silver

**Affiliations:** 1 Case Western Reserve Univ., Dept. of Neurosciences, 10900 Euclid Ave., SOM E654, Cleveland, OH, 44106, USA; 2 Athersys, Inc. Regenerative Medicine, Cleveland, OH, 44115, USA; 3 ReGenesys, Bioincubator Leuven, 3001, Leuven, Belgium.

## Abstract

Following spinal cord injury (SCI), immune-mediated secondary processes exacerbate the extent of permanent neurological deficits. We investigated the capacity of adult bone marrow-derived stem cells, which exhibit immunomodulatory properties, to alter inflammation and promote recovery following SCI. *In vitro,* we show that human multipotent adult progenitor cells (MAPCs) have the ability to modulate macrophage activation, and prior exposure to MAPC secreted factors can reduce macrophage-mediated axonal dieback of dystrophic axons. Using a contusion model of SCI, we found that intravenous delivery of MAPCs one day, but not immediately, after SCI significantly improves urinary and locomotor recovery, which was associated with marked spinal cord tissue sparing. Intravenous MAPCs altered the immune response in the spinal cord and periphery, however biodistribution studies revealed that no MAPCs were found in the cord and instead preferentially homed to the spleen. Our results demonstrate that MAPCs exert their primary effects in the periphery and provide strong support for the use of these cells in acute human contusive SCI.

Spinal cord injury (SCI) and resulting paralysis is a critical unmet medical need worldwide. Neurological deficits following traumatic SCI are severe and often permanent^1^,^2^,^3^,^4^ due to the loss of ascending and descending axonal pathways, demyelination, and lack of substantial axonal regeneration and plasticity^5^,^6^,^7^,^8^. Following the primary mechanical insult, a cascade of cellular and molecular events occur over the course of weeks causing destruction of initially spared spinal cord tissue^9^. This process includes the breakdown of the blood brain barrier and subsequent infiltration of immune cells to the injury site and surrounding parenchyma^10^. The influx of immune cells into the injured CNS has largely been considered to be detrimental, but recent studies have shown the effects of this process to be complex and often critical to meaningful repair, especially at later stages^11^,^12^,^13^. Macrophages, the dominant infiltrating cell type, can exacerbate secondary injury acutely by causing axonal dieback, but also promote repair chronically by clearing debris and promoting remyelination, depending on the microenvironment and their activation state^14^,^15^,^16^,^17^,^18^,^19^,^20^. Targeting immune-mediated secondary injury soon after the primary injury offers a therapeutic avenue to treat traumatic SCI^21^.

Multipotent adult progenitor cells (MAPCs) are a well characterized population of adherent non-hematopoietic cells isolated from adult bone marrow^22^,^23^. MAPCs exhibit a distinct gene expression profile when compared to mesenchymal stem cells and they maintain stable gene expression and karyotype across multiple passages^23^,^24^,^25^. Clinical-grade MAPCs have been isolated under similar conditions and are currently active in phase II clinical development as an “off-the-shelf” allogeneic infused cell product for treatment of ischemic stroke^26^. MAPCs have strong immunomodulatory properties and promote tissue regeneration and functional recovery in neurological, cardiovascular, and immunological disorders^23^,^25^,^27^,^28^,^29^,^30^,^31^,^32^,^33^. We previously demonstrated that intraparenchymal injection of rat MAPCs attenuated macrophage-mediated axonal dieback in a dorsal column crush model of spinal cord injury^28^, however the long term functional benefits in a clinically relevant contusion injury model were not explored.

Here we show that a single intravenous dose of human MAPCs delivered one day following contusive SCI remarkably improves both locomotor and urinary functions. Interestingly, MAPCs rarely enter the nervous system, however, a significant increase of white matter sparing and a marked change in macrophage/microglia activation is observed in the spinal cord. This study highlights the promise of therapies directed at altering the peripheral response to immune-mediated CNS pathologies and establishes human MAPCs as a novel therapy for the treatment of acute SCI.

## Results

### Intravenous human MAPCs enhance locomotor recovery after SCI

Contusive injury is the most common form of SCI affecting humans^34^. We modeled this injury in rodents using the Infinite Horizon device, delivering a controlled impaction (250 kDyne) to the spinal cord at thoracic level 8. This reproducible injury has been shown to accurately represent primary and secondary injury cascades as well as permanently impair both locomotor and urinary behaviors^35^,^36^,^37^.

**Figure 1:**
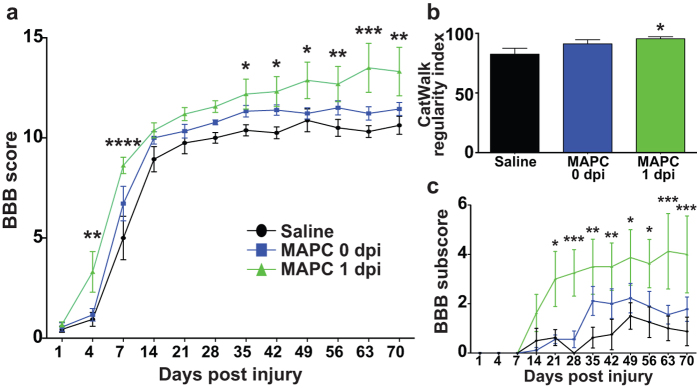
MAPC treatment one day post injury improves locomotor recovery. The (**a**) BBB scale and (**c**) BBB subscale of rats treated with Saline, MAPC 0 dpi or MAPC 1 dpi over a 10 weeks postoperative period. *n* = 8 Saline, 9 MAPC 0 dpi, 8 MAPC 1 dpi. *p < 0.05, **p < 0.01, ***p < 0.001, ****p < 0.0001 when compared to Saline treated. Two-way repeated measures ANOVA Fisher’s Least Significant Difference post-hoc test. (**b**) Interlimb coordination at 10 weeks post injury as measured by CatWalk regularity index. *n* = 7 Saline, 8 0 dpi , 8 1 dpi. *p < 0.05 when compared to Saline treated. Kruskal-Wallis ANOVA with Dunn’s post-hoc test. Data represent mean and s.e.m.

We examined locomotor recovery following contusion SCI using the Basso, Beattie and Bresnahan (BBB) scale^38^. Immediate intraspinal injection of human MAPCs did not effectively promote locomotor recovery (Supplemental Fig. 1). Intraspinal delivery circumvents peripheral immune organs, which hold reserves of monocytes and other immune cells that mobilize in response to the injury. Systemic intravenous treatment with human MAPC has shown efficacy in reducing infarct size, closing the blood brain barrier, and hindering secondary injury cascades in multiple neurological indications, including traumatic brain injury and ischemic stroke^31^,^33^. Thus, we examined intravenous delivery of human MAPCs for treatment of SCI. Animals received an intravenous dose of 4 million MAPCs immediately following injury (MAPC 0 dpi), one day post injury (MAPC 1 dpi), or saline control following contusive SCI. Vehicle treated animals recovered from hindlimb paralysis to an average BBB score of 10.63 at 70 dpi, which corresponds to the ability to take an occasional hindlimb step (Fig. 1a). Intravenous delivery of MAPCs immediately following the injury did not significantly improve locomotor outcomes (Fig. 1a). However, MAPC treatment 1 day post-injury led to significant improvements in locomotion (Fig. 1a). Animals improved to an average BBB score of 13.31 at 70 dpi, corresponding to consistent hindlimb stepping with frequent coordination between the four limbs. Interestingly, significant locomotor improvements were observed by 4 dpi and were maintained throughout the experiment, suggesting a strong neuroprotective effect. Regenerative therapies typically demonstrate a more delayed response^37^,^39^,^40^,^41^,^42^. Inter-limb coordination was confirmed using the CatWalk regulatory index, an unbiased approach to measuring coordinated locomotion^43^ (Fig. 1b). Additionally, significant improvements were observed in the BBB subscore^44^, which evaluates the fine details of locomotion (Fig. 1c). Importantly, no signs of hyperalgesia were present in any treatment group, as all animals had similar responses to heat stimuli (Supplemental Fig. 1).

### MAPCs promote recovery of micturition

**Figure 2:**
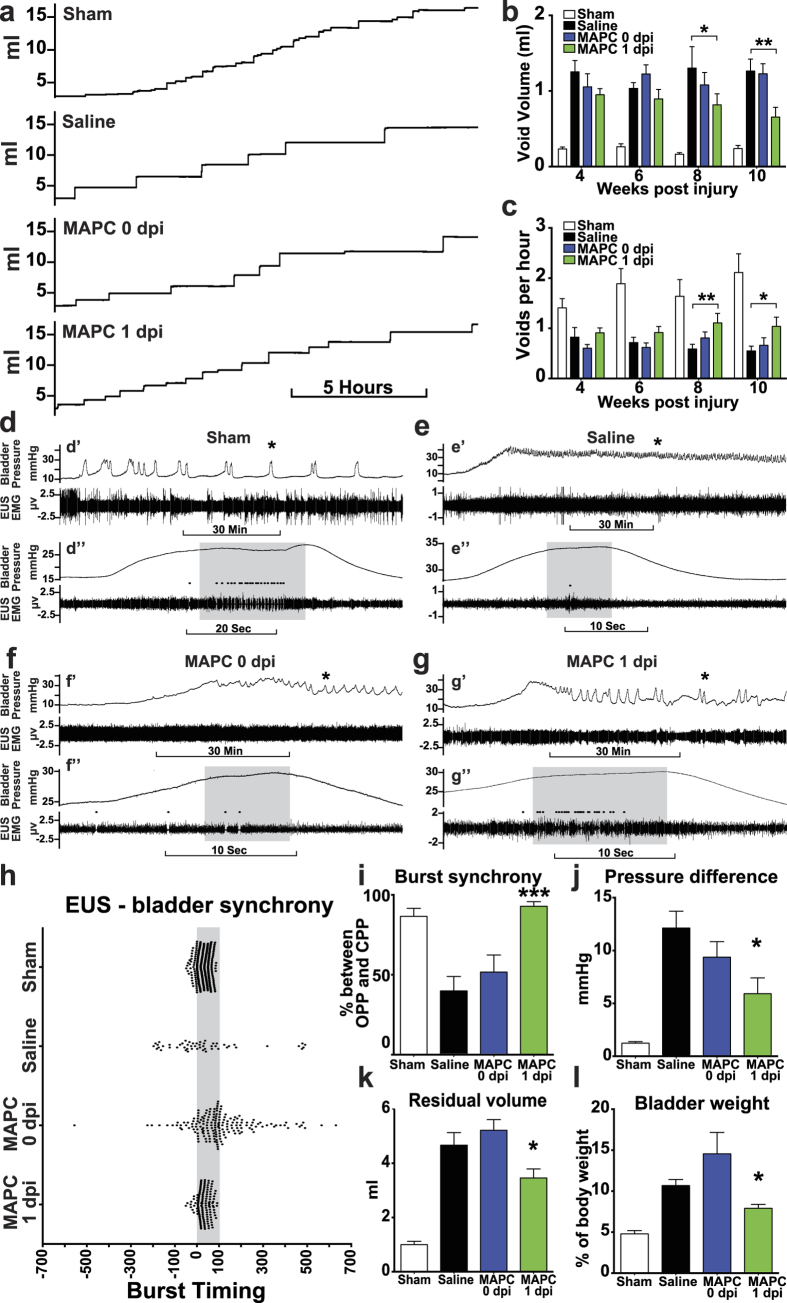
MAPC treatment one day post injury improves micturition pattern and lower urinary tract coordination. (**a**) Representative smoothed metabolic cage traces 10 weeks after injury of a Sham, Saline treated, MAPC 0 dpi treated, and MAPC 1 dpi treated animals. (**b,c**) Metabolic cage quantification of average void frequency and void volume. *n* = 6 Sham, 8 Saline, 9 MAPC 0 dpi, and 8 MAPC 1 dpi. *p < 0.05, **p < 0.01 when compared to Saline treated. Two-way repeated measures ANOVA, Fisher’s Least Significant Difference post-hoc test. (**d–g**) Representative urodynamic recordings at 10 weeks after SCI. (d’–g’) Compressed view of bladder pressure (top trace) and EUS EMG activity recordings (bottom trace). Asterisks mark expanded void shown below. (d”–g”) Expanded time points from d’–g’. Grey box denotes the time between OPP and CPP. Black dots denote EUS bursting. (**h**) Scatter plot of EUS bursting over three void cycles in relation to bladder contractions. Grey box denotes the time interval between OPP and CPP (0%–100%). (**i**) Percentage of EUS bursts between OPP and CPP of bladder contractions. (**j**) Average bladder pressure difference from baseline to post void. (**k**) Average residual bladder volume following a void. (**l**) Bladder weight 10 weeks post injury. *n* = 5–6 Sham, 8 Saline, 9 MAPC 0 dpi, 8 MAPC 1 dpi. *p < 0.05, ***p < 0.001 when compared to Saline treated. One-way ANOVA, Fisher’s Least Significant Difference post-hoc test. Data represent mean and s.e.m.

SCI disrupts the connections between the brainstem micturition control center and the lower urinary tract, leading to impaired urination^4^. In humans, this disruption in urinary control is treated by catheterization, which severely lowers quality of life and contributes to frequent urinary tract infections. In rodents, voluntary micturition is lost for the first few weeks following SCI, when bladders have to be expressed manually. As spinal shock subsides, a small amount of function is restored, though void frequency and volume are significantly altered. Similar to locomotor recovery, intraspinal or intravenous MAPC 0 dpi did not improve urinary function as measured by a metabolic cage system (Supplemental Fig. 1, Fig. 2a–c). However, intravenous MAPC treatment 1 dpi significantly improved void volume and frequency 8 and 10 weeks post injury, with trending improvements observed as early as week four (Fig. 2a–c).

To investigate the recovery of coordination between muscles controlling micturition we utilized terminal urodynamic analyses under light urethane anesthesia. During a normal void, the external urethral sphincter (EUS) phasically bursts in coordination with a detrusor contraction, causing a rapid increase in bladder pressure and urine expulsion. Sham operated animals demonstrated coordination between bladder contractions and EUS bursting patterns, with most bursts occurring between the opening peak pressure (OPP) and closing peak pressure (CPP) (Fig. 2d,h,i, Supplemental Fig. 2). Following SCI, the detrusor becomes hyperactive and contracts the bladder against a closed, irregularly-bursting EUS, resulting in detrusor sphincter dyssynergia. This is demonstrated by a large decrease in the number of bursts (Supplemental Fig. 2), coupled with little coordination between EUS bursting and bladder contractions (Fig. 2e,h,i, Supplemental Fig. 2). Conversely, the length of a bladder contraction (Supplemental Fig. 2) and time between the opening and closing peak pressure (Supplemental Fig. 2) were not affected by injury or treatment, nor was the basic ability to burst (Supplemental Fig. 2), as most non-treated injured animals still displayed uncoordinated bursting (Supplemental Fig. 2). MAPC treatment at 1 dpi increased the number of EUS bursts three fold, and led to proficient coordination between the EUS and detrusor (Fig. 2g–i). MAPC treatment did not alter the pressure at which a void was initiated (Supplemental Fig. 2), or the peak pressure of a void (Supplemental Fig. 2). However, the pressure following a void was significantly lower and closer to baseline (Fig. 2j) and the residual volume of urine after voiding was significantly decreased by MAPC treatment (Fig. 2k), suggesting that the efficiency of voids was also improved. Interestingly, MAPCs given at 0 dpi also increased bursting similar to MAPCs at 1 dpi (Supplemental Fig. 2), however bursting was not synchronized with bladder contractions and voiding was inefficient (Fig. 2f,h–k), illustrating the importance of bladder-EUS coordination in promoting void efficiency. Following SCI the bladder enlarges and distends to increase capacity due to inefficient voiding. MAPC treatment 1 dpi decreased the volume at which a void was initiated (Supplemental Fig. 2) and decreased bladder weight (Fig. 2l), which is indicative of a healthier bladder. Together, these data suggest that MAPC treatment 1 dpi restores bladder-EUS synchrony, resulting in more efficient voids with a smaller, healthier bladder.

### MAPCs exert benefit in a dose dependent manner

To optimize MAPC treatment, we performed an *in vivo* dose response study. Animals were treated with either 100,000, 400,000, 1 million, 4 million or 8 million MAPCs at 1 dpi, with functional readouts analyzed through week 10. Both the 4 million and 8 million doses significantly and similarly improved locomotion (Supplemental Fig. 3) and void frequency (Supplemental Fig. 3) over lower doses. Significant improvements in the BBB subscore were also observed with 1 million MAPCs (Supplemental Fig. 3), however, void volume showed a non-significant (p = 0.0711) trend at 4 million MAPCs (Supplemental Fig. 3). This suggests MAPCs act in a dose dependent manner, with 4 million cells being optimal for efficacy in our rat model of contusive SCI.

### MAPCs enhance tissue sparing and decrease astroglial reactivity

**Figure 3:**
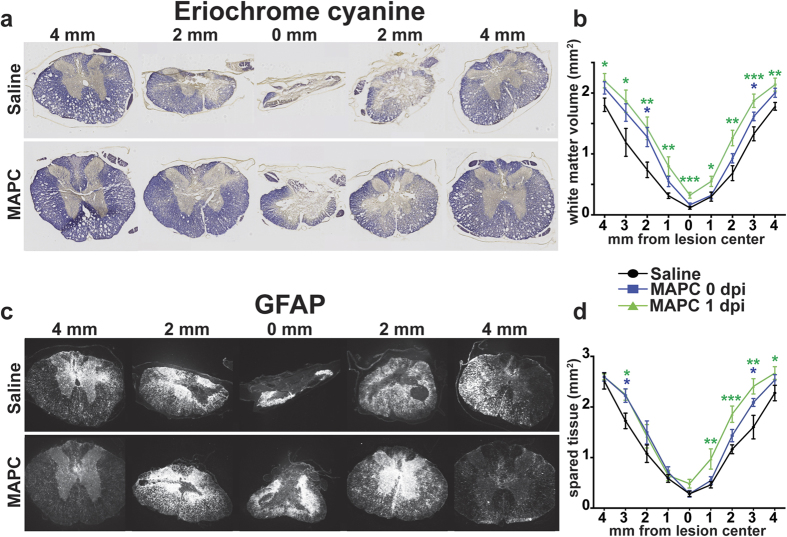
MAPCs promote spinal cord tissue sparing. (**a**) Representative Eriochrome cyanine staining of spared white matter ten weeks after SCI. (**b**) Quantification of white matter sparing ten weeks after SCI. *n* = 8 Saline, 9 MAPC 0 dpi, 8 MAPC 1 dpi. (**c**) Representative GFAP immunostaining ten weeks after SCI. (**d**) Quantification of the spared spinal cord tissue ten weeks after SCI. *n* = 8 Saline, 9 MAPC 0 dpi, 7 MAPC 1 dpi. *p < 0.05, **p < 0.01, ***p < 0.001, ****p < 0.0001 when compared to Saline treated. One-way ANOVA, Fisher’s Least Significant Difference post-hoc test. Data represent mean and s.e.m.

The secondary injury cascade triggered by SCI leads to the formation of a large cavity, which spreads in both rostral and caudal directions from the lesion epicenter. We quantified the lesion cavity with two measures: Eriochrome cyanine (EC) staining of intact white matter (Fig. 3a), and GFAP immunofluorescence of reactive astrocytes (Fig. 3c). MAPC treatment at 1 dpi resulted in a significant amount of spared white matter (Fig. 3b) and tissue (Fig. 3d) at the epicenter and continued several segments in both rostral and caudal directions. Reduced astrocyte reactivity was observed in MAPC treated animals in areas distal to the lesion, but unchanged in the lesion center (Supplemental Fig. 4). Interestingly, we also observed some white matter and tissue sparing in animals that received MAPCs immediately following injury, which did not lead to functional improvements. Importantly, however, the significant sparing following immediate treatment was mainly observed rostral and caudal to the epicenter, not in the lesion core.

### MAPCs modulate the acute inflammatory response to SCI

**Figure 4:**
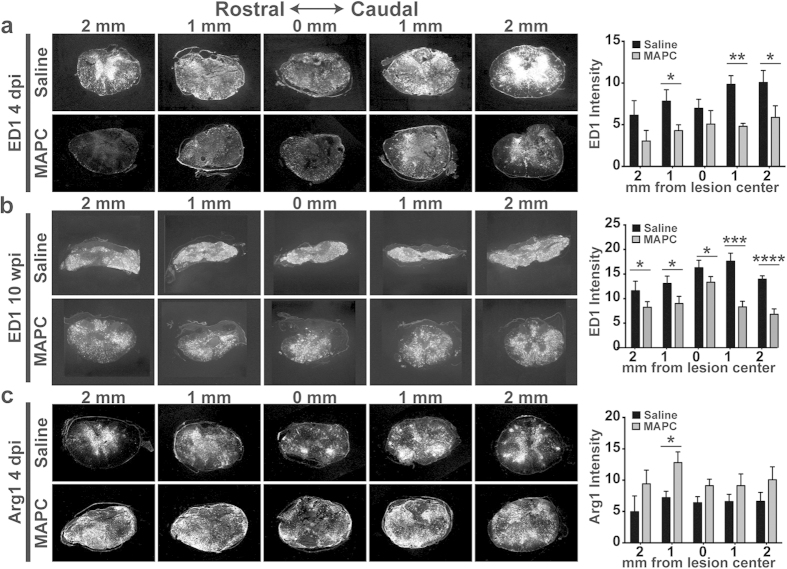
Intravenous MAPCs reduce inflammatory macrophages in the spinal cord after SCI. (**a**) Representative images and quantification of ED1 immunostaining of spinal cord tissue in Saline and MAPC 1 dpi treated groups four days after SCI. *n* = 6 per group. *p < 0.05, **p < 0.01, Student’s t-test (**b**) Representative images and quantification of ED1 immunostaining of spinal cord tissue in Saline and 1 dpi MAPC treatment ten weeks after SCI. *n* = 8 per group. Error bars show s.e.m. *p < 0.05, ***p < 0.001, ****p < 0.0001, Student’s t-test (**C**) Representative images and quantification of Arg1 immunostaining of spinal cord tissue in Saline and 1 dpi MAPC treatment four days after SCI. *n* = 6 per group. Error bars show s.e.m. *p < 0.05, Student’s t-test. Data represent mean and s.e.m.

Preservation of tissue at the injury site suggests that MAPC treatment may alter the inflammatory response following injury. We observed a significant reduction in ED1+ macrophages/microglia in MAPC treated animals at 4 dpi, which was maintained through 10 weeks post injury (Fig. 4a,b). Further, we observed a significant increase of Arginase 1 (Arg1) positive macrophages (Fig. 4c), a well-established marker of alternatively activated and tissue-healing macrophages, at 4 dpi in MAPC treated animals.

**Figure 5:**
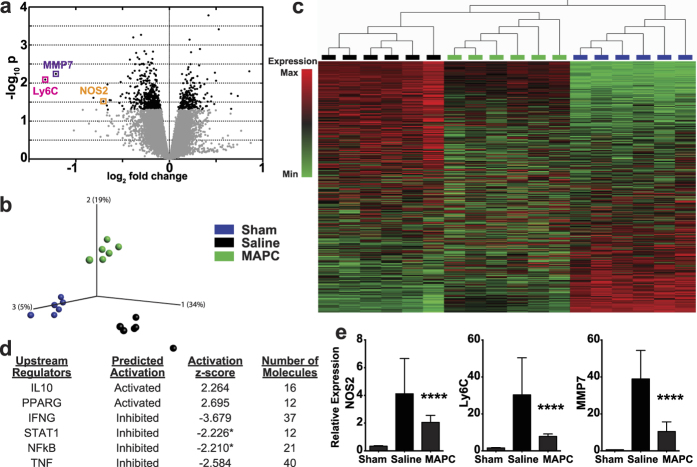
Microarray analysis reveals that MAPC treatment 1 dpi modulates immune related genes and pathways at the injury site four days post SCI. (**a**) Volcano plot showing differentially expressed genes at the injury site following MAPC treatment compared to saline treatment. Black circles p < 0.05, grey circles p > 0.05, t-statistic with Bayesian adjusted denominator (**b**) Principal component analysis of each individual animal’s expression of genes significantly modulated by MAPC treatment compared to saline treatment. (**c**) Dendrogram of unsupervised agglomerative hierarchical cluster analysis and gene expression heat map of genes significantly modulated by MAPC treatment compared to saline treatment. (**d**) Upstream regulators significantly modulated by MAPC treatment using Ingenuity pathway analysis. * denotes a bias corrected z-score. (**e**) Real-time qPCR verification of NOS2, Ly6C, and MMP7 gene expression changes. *n* = 6 per group, 3 biological replicates per animal. ****p < 0.0001 when compared to Saline treatment. Two-way ANOVA, Fisher’s Least Significant Difference post-hoc test.

To further investigate MAPC-induced modulation of the acute inflammatory response to SCI, we performed a RNA microarray at the injury site 3 days following intravenous saline or cell administration at 1 dpi. Compared to the non-injured sham, injury plus saline treatment induced a significant change in over 14,000 genes, of which approximately 50% were upregulated (Supplemental Fig. 5). In contrast, MAPC treatment significantly modulated 698 genes when compared to saline treatment (Fig. 5a), significantly down regulating 66.2% of these genes. Principal component analysis of significant MAPC-modulated genes uncovers three distinct clusters corresponding to the treatment groups (Fig. 5b), with unsupervised hierarchical clustering and gene expression heat map revealing MAPC treated animals’ gene expression to be intermediate between saline and sham (Fig. 5c). Functional annotation of the MAPC-modulated gene set by Ingenuity Pathway Analysis (Qiagen) predicted that many of the expression changes can be attributed to activation of upstream regulators including interleukin 10 (IL-10) and peroxisome proliferator-activated receptor gamma (PPARG) (Fig. 5d), known positive regulators of alternatively activated macrophages^45^,^46^,^47^. There was also inhibition of known activators of pro-inflammatory macrophages including interferon gamma (IFNG)^48^, signal transducer and activator of transcription 1 (STAT1)^49^, nuclear factor kappa B (NFkB)^50^, and tumor necrosis factor (TNF)^51^ (Fig. 5d). Functional annotation indicated that the changes in gene expression led to a decrease in phagocyte migration (12 molecules, p = 0.0171) and modulated leukocyte activation (27 molecules, p = 0.0122), especially those genes associated with monocyte activation (21 molecules, p = 0.0042). qPCR confirmation of differentially expressed genes detected by the microarray was consistent with decreased inflammation at the injury site. The macrophage marker Ly6C was substantially reduced at the lesion epicenter as compared to control (Fig. 5e). Further, MAPC treatment reduced matrix metalloproteinase-7 (MMP7) (Fig. 5e), which has been shown to facilitate immune access to the CNS^52^. Additionally, expression of the classically activated macrophage marker inducible nitric oxide synthase (NOS2) was reduced (Fig. 5e).

Following SCI, the majority of infiltrating monocytes originate from the spleen^53^. We therefore examined by qPCR the spleen and whole blood for gene changes to support and extend our observations in the spinal cord. A reduction in NOS2 was observed in the blood (Supplemental Fig. 5) and spleen (Supplemental Fig. 5) following MAPC treatment. Consistent with the predicted inhibition in IFNG signaling in the spinal cord, we detected a significant decrease in the pro-inflammatory cytokine interferon gamma-induced protein 10 (CXCL10) in the blood (Supplemental Fig. 5) and interferon stimulated genes 54 and 60 (ISG54 and ISG60) in the spleen (Supplemental Fig. 5). ISG54 and ISG60 reductions are also consistent with the inhibition of TNF as an upstream regulator. Interestingly, expression of the T regulatory cell (Treg) marker Foxp3 was considerably increased in the blood (Supplemental Fig. 5), as were the negative co-stimulatory T cell receptors cytotoxic T-lymphocyte-associated protein 4 (CTLA4) in the blood and spleen (Supplemental Fig. 5) and lymphocyte-activation gene 3 (LAG3) in the spleen (Supplemental Fig. 5). These genes are associated with immune-suppressive functions^54^, suggesting that MAPC modulation of T cells may play a role in recovery. Further work is needed to elucidate the complex interactions of these immune populations after treatment with MAPCs.

### MAPCs do not home to the site of spinal cord injury

**Figure 6:**
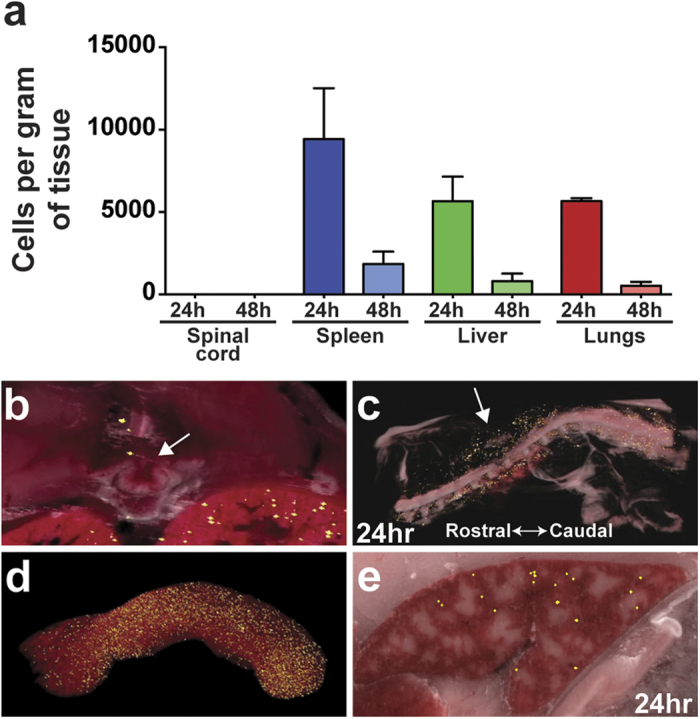
Biodistribution of intravenously administered MAPC. (**a**) Number of MAPCs per tissue weight at 24 or 48 hours after i.v. injection of 4 × 10^6^ cells at one dpi. *n* = 3 per group, except *n* = 2 for 24 hour liver. Data represent mean and s.e.m. (**b**) Biodistribution of Qdot labeled cells around the lesion. Note no cells are found in the spinal cord. Arrow denotes the dorsal injured cord. (**c**) Representative 3D overlaid fluorescent/bright field image of the spinal column 24 hours following MAPC injection. Arrow denotes the dorsal injury location. (**d**) 3D Representative fluorescent CryoViz image of Qdot labeled cells in the spleen 24 hours following MAPC injection. (**e**) Representative bright field CryoViz image of Qdot labeled cells of a single plane in the spleen 24 hours following MAPC injection.

The fact that intravenous delivery of MAPCs modulate immune-related genes in the periphery and decrease leukocyte activation and migration into the spinal cord suggests that the cells may manifest their therapeutic effects in one or more peripheral organs. Therefore, we sought to determine the *in vivo* biodistribution of MAPC following SCI. We injected quantum dot labeled MAPCs intravenously at 1 dpi and visualized the cells 24 or 48 hours later using CryoViz technology^55^ (BioInVision). Surprisingly, no MAPCs were found in the injured cord (Fig. 6a,b, Supplemental Movie 1), although cells appeared to home to areas of peripheral tissue damage dorsal to the SCI site (Fig. 6b,c). Cell numbers decreased markedly from 24 to 48 hours in all other tissues (Fig. 6a, Supplemental Fig. 6). A large number of cells were found within filtering organs, such as the lungs and liver, with the greatest number of cells per tissue weight observed in the spleen (Fig. 6a,d, Supplemental Fig. 6). MAPCs were found predominantly near the marginal zone in the red pulp (Fig. 6e), demonstrating the possibility for an interaction with the splenic leukocyte population. Homing of MAPC to the spleen in a dose dependent manner following intravenous administration one day after traumatic brain injury has been previously reported^27^. Following SCI, the spleen decreases in weight and size due to splenic leukocyte apoptosis^56^,^57^ and a large efflux of monocytes migrating to areas of inflammation and injury^53^. MAPC treatment preserved splenic mass suggesting treatment may affect the survival and/or migration of splenocytes (Supplemental Fig. 6).

### Secreted MAPC factors alter macrophage phenotype and reduce axonal dieback

**Figure 7:**
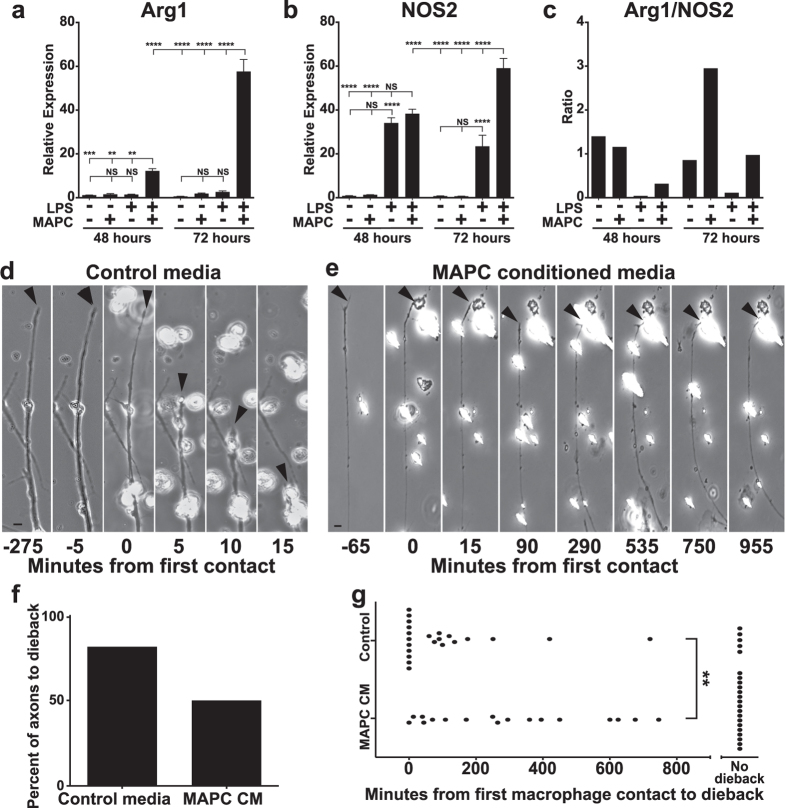
MAPCs alter macrophage expression of Arginase1 and NOS2 and hinder macrophage mediated axonal dieback *in vitro*. (**a,b**) Macrophage expression of Arg1 and NOS2 following LPS and/or MAPC treatment as measured by qPCR and normalized to beta actin. *n* = 6 per group. *p < 0.05, **p < 0.01, ***p < 0.001, ****p < 0.0001. One-way ANOVA, Fisher’s Least Significant Difference post-hoc test. Data represent mean and s.e.m. (**c**) Ratio of Arg1/NOS2 expression following LPS and/or MAPC treatment as measured by qPCR and normalized to beta actin. (**d**) Representative time-lapse images of rapid axonal dieback from macrophages pretreated with control media. Arrowhead denotes tip of axon. (**e**) Representative time-lapse images of the failure of macrophages pretreated with MAPC conditioned media to induce axonal dieback. Arrowhead denotes tip of axon. (**f**) Percent of dystrophic axons that underwent dieback following contact with a macrophage. *n* = 27 control, 34 MAPC-CM. (**g**) Minutes lapsed from a macrophage’s first contact with a dystrophic axon to dieback. **p < 0.01, Student’s t-test. Scale bar = 10 μm.

The increased Arg1+ staining in the spinal cord and decreased NOS2 expression in all tissues examined suggests that MAPCs can modulate the activation state of macrophages. We therefore tested whether MAPCs could alter macrophage activation directly (Supplemental Fig. 7). Cultured cell line (NR8383) rat macrophages expressed low levels of NOS2 and Arg1. However, within 48 hours of lipopolysaccharide (LPS) stimulation NOS2 expression increased more than 40 fold while Arg1 expression remained unchanged (Fig. 7a,b). Culturing MAPCs in transwell with unstimulated macrophages had little effect on macrophage NOS2 or Arg1 expression (Fig. 7a,b). However, if macrophages were first stimulated with LPS, MAPCs increased macrophage Arg1 expression greater than 20 fold (Fig. 7a). We also observed a 2.5 fold increase in macrophage NOS2 expression following MAPC treatment (Fig. 7b). Importantly, MAPC treatment shifted the ratio of macrophage activation markers Arg1 and NOS2 strongly towards an alternatively activated, anti-inflammatory phenotype (Fig. 7c).

Taken together with previous data in other models of CNS injury^27^,^32^,^33^, this result supports the intriguing hypothesis that MAPC-mediated tissue sparing is not due to a direct interaction at the spinal cord injury site, but an indirect effect through modulation of infiltrating immune cells from the periphery. Previous studies from our laboratory have demonstrated the direct role of activated peripheral macrophages in axonal dieback following SCI^58^,^59^. To examine the effects of MAPCs on macrophage-mediated axonal dieback we utilized an *in vitro* model of the glial scar known as the spot assay, in which neurons form dystrophic endballs as they attempt to traverse an increasing gradient of chondroitin sulfate proteoglycans^37^,^60^. Here we cultured adult sensory neurons in the spot assay, and added NR8383 macrophages after baseline observation using time-lapse microscopy (Supplemental Fig. 7). Because intravenously delivered MAPCs did not penetrate the CNS, we investigated the ability of these cells to modulate the downstream activity of macrophages by first pretreating macrophages with MAPC conditioned media (MAPC-CM) for 24 h prior to washing and coculturing with neurons on the spot assay (Supplemental Fig. 7). When control media pre-treated macrophages contacted a dystrophic axon, the axon underwent rapid dieback over 80% of the time (Fig. 7d,f,g). However, MAPC-CM pre-treated macrophages frequently failed to cause dieback in dystrophic axons (Fig. 7e,f). Further, when dieback did occur it was significantly delayed (Fig. 7g, Supplemental Fig. 7). This data suggests that prior exposure to MAPC secreted factors can modulate the ability of macrophages to induce axonal dieback.

## Discussion

The majority of contusive injuries to the spinal cord are incomplete and leave behind intact remnants of white matter tracts. No clinical treatments exist to ameliorate the progressive immune-mediated secondary damage to these tracts that rapidly follows the primary mechanical impact. The results of this study show that a single intravenous administration of human MAPCs, with a clinically relevant delay of one full day, following contusive spinal cord injury leads to marked tissue sparing, especially in white matter at the lesion epicenter and beyond, with accompanying improvements in locomotor and bladder function.

We found no evidence for MAPCs within the spinal cord injury site, and instead MAPCs homed to damaged tissue peripheral to the injury site and to the spleen in greater numbers than filtering organs such as the lungs and liver. Blomster *et al.* showed that acutely following SCI the majority of infiltrating monocytes originate from the spleen and are Ly6C^high^ 53. The preservation of splenic mass, along with the reduction of ED1+ cells and Ly6C expression in the vicinity of the injury, suggest that MAPCs may inhibit splenic monocyte mobilization. Further, gene changes detected by microarray analysis predicted a decrease in phagocyte migration. Those monocytes that do mobilize may then have an altered phenotype, as we saw a significant decrease in NOS2 expression in the spleen, blood, and SCI site, as well as an increase in Arg1+ in the spinal cord. Monocyte infiltration into the injured CNS could also be modulated by MAPC-facilitated blood brain barrier repair^27^, although a recent study suggests that following SCI, blood brain barrier breakdown contributes little to macrophage infiltration^61^. Thus, monocyte extravasation appears to be an active process. Splenic mass may also be preserved through inhibition of apoptosis or an increase in proliferation^27^. Overall, these data suggest that MAPCs exert their primary effects in the periphery and not via direct interactions in the injured spinal cord.

Macrophages are known to have a spectrum of activation states that can cause additional CNS damage or aid in repair. However, the neurotoxic phenotype typically overwhelms the reparative after SCI^14^. We show here that MAPC treatment may alter both the activation state and total number of macrophages, as we observed a transient increase in Arg1+ and a sustained decrease of ED1+ immunostaining in the spinal cord following injury. Previous studies have shown a shift in macrophage activation following intravenous MAPC treatment in traumatic brain injury^33^. Our novel microarray data is consistent with gene changes expected in an attenuated inflammatory response and predicted a decrease in activation. Culturing macrophages in transwell with MAPCs shifted macrophages towards an anti-inflammatory phenotype. Further, treating macrophages with MAPC conditioned media curtails macrophage-mediated axonal dieback, suggesting that previous exposure to MAPC-secreted factors outside the CNS could potentially lead to lasting changes in polarization that impact the outcome of these interactions.

We also observed increases in Foxp3, a Treg marker, and the negative co-stimulatory T cell receptors CTLA4 and LAG3, suggesting that the T cell response is positively modulated with MAPC treatment. CNS antigen recognizing T cells and Tregs are crucial for promoting neuronal survival and recovery, and ablation of Tregs following injury has detrimental effects^62^,^63^. We have previously observed induction of CD4+/CD25+/FOXP3+ Treg cells following MAPC treatment *in vitro*^64^ and *in vivo*^33^. MAPCs may also be affecting macrophage phenotype indirectly through T cell modulation. Tregs are known to have strong anti-inflammatory properties that can alter macrophage invasion and activation, and can prevent secondary infarct growth by counteracting excessive production of pro-inflammatory cytokines following CNS injury^65^,^66^, however further investigation is warranted to elucidate the role of MAPCs in T cell activation following spinal cord injury.

It is evident that the timing of intravenous MAPC delivery is important. Physiological recovery was considerably greater when delaying the treatment by one day. Multiple doses over the course of many days may confer additional benefit, though our data suggests that increasing a single dose beyond 4 million cells in this model does not increase functional recovery. This dose was also found to be optimally effective when administering MAPC intravenously one day after induction of ischemic stroke^67^. The number of cells in circulation decreases rapidly, so timing MAPC treatment with the peak inflammatory response is most likely critical. For example, adaptive transfer experiments show that splenic monocyte recruitment to the injury site does not occur until several days following injury^53^. MAPCs immediately administered following injury would most likely be cleared prior to the onset of recruitment signaling, however delaying treatment by one day would allow MAPCs to be present in the spleen at signaling onset. Our working hypothesis is that MAPC will be most effective at this time because the cells will be exposed to proinflammatory cytokines and chemokines which will likely alter their homing and cytokine production. This concept of licensing by the proinflammatory microenvironment has been well established^68^. Consistent with this hypothesis, MAPCs had much greater influence on LPS-stimulated macrophages compared to those that were non-stimulated.

Immunosuppression may enhance the survival of MAPC, however, because their mechanism of action is immunomodulatory, this would likely confound our results and ultimately dampen efficacy. To circumvent systemic immune surveillance, MAPCs can be injected directly into the spinal cord. Others have reported efficacy using mesenchymal stem cells^69^, and it is possible that further studies are needed to optimize this route of administration for MAPC.

A major concern among the spinal cord injured population is urinary tract control^70^ and, importantly, we observed dramatic improvements in EUS-detrusor synchrony following our acute stem cell administration strategy. MAPC treatment 1 dpi lead to improvements in the quality of daily voiding patterns, diminished sphincter-detrusor dyssynergia, and enhanced voiding efficiency. Interestingly, all injured groups showed similar detrusor activity globally, as the void initiation pressure, max void pressure, and void duration were comparable. However, MAPC 1 dpi treated animals recovered EUS-detrusor coordination allowing for greater void efficacy, resulting in a lower baseline bladder pressure and residual volume, and a smaller bladder. Bladder-EUS coordination is dependent on white matter sparing^71^, which was a consistent effect of the 1 dpi MAPC treatment.

Our results demonstrate that intravenous MAPCs exert their primary effects in the periphery, and suggest that modulation of leukocytes may be highly effective in altering the course of immune-mediated secondary pathologies after spinal cord injury. Taken together, our work suggests a conserved mechanism of MAPC action across a number of CNS injury models involving the peripheral immune system, resulting in mitigation of adverse aspects of inflammation and modulation of macrophages and T cells towards a reparative phenotype. The clinical-grade version of the cell therapy tested here has been shown to be safe in numerous early stage clinical trials and is currently in clinical development for ischemic stroke^26^. These data provide strong support for the use of MAPCs for acute treatment of adult contusive SCI patients.

## Methods

### Study design

This controlled laboratory experiment was designed to test if human MAPCs can improve locomotor and bladder function in rats with severe, incomplete spinal cord injury, and explore the mechanism by which these cells may confer benefit. One hundred fifty-nine Sprague Dawley rats, weighing 225-249 g, were randomly assigned to MAPC or saline treatment groups (Table 1). Treatment was administered either intravenously or intraspinally immediately following spinal cord injury, or intravenously one day following injury. Locomotor assessment was evaluated by the BBB open field locomotor test preformed at days 1, 4, 7, and weekly thereafter until 6 or 10 weeks post injury. The CatWalk locomotor analysis was performed on one cohort of animals at 10 weeks post injury. Bladder assessment was performed by metabolic cage analysis at weeks 4, 6, and 10 weeks post injury. Urodynamic analysis was performed on one cohort of animals at 10 weeks post injury, followed by sacrifice and histological investigation. A second group of 30 animals were treated by intravenous MAPC or saline treatment one day post injury and sacrificed at 4 days post injury, and underwent microarray analysis and qPCR confirmation, or histological analysis. Another group of 7 animals received Qdot labeled MAPCs to visualize biodistribution. *In vitro* macrophage activation and behavior following MAPC treatment was investigated with the rat macrophage line NR8383, as these macrophages display classic activation like behavior and induce axonal dieback. Animals were immediately excluded from the study if surgical complications arose, such as failed intravenous injections or abnormal contusion hits as defined by the force/displacement graph of the Infinite Horizon Impactor (n = 35). Animals were excluded from the study if they developed bladder infections or other health complications (n = 8). All animals that reached the predetermined endpoints are present in this study. One sham animal failed to record EUS-EMG activity during urodynamic assessment and was not included in bursting analysis. A statistical outlier was identified in the CatWalk regularity index data by ROUT analysis and was excluded from this analysis (MAPC 0 dpi, CatWalk regularity index = 29.63). Animal caretakers and behavioral investigators were blind to the treatment. Rats with different treatments were housed together, and blind observers using objective readouts acquired and analyzed all data.

### Rat macrophage culture

NR8383 (ATCC) macrophages, an adult Sprague Dawley alveolar cell line, were stored in liquid nitrogen. Cells were thawed quickly in 37 °C water bath and transferred to 15 ml conical tube containing 10 ml of pre-warmed NR8383 media (F-12 (Gibco/Life Technologies) supplemented with penicillin/streptomycin (Invitrogen) and fetal bovine serum (Invitrogen)), centrifuged at 300 × g for 7 minutes, supernatant removed, and resuspended in NR8383 media. Cells were cultured in uncoated tissue culture flasks (Corning) at 5% CO_2_ for seven days, and media was changed every 2–3 days.

### Macrophage and MAPC cocultures

NR8383s were cultured as described above, harvested with trypsin/EDTA (Invitrogen), washed three times, and plated in uncoated 24 well plates at 200,000 cells in 0.6 ml per well, with some receiving 100 ng LPS (Invivogen), and cultured at 5% CO_2_ for 24 hours. Frozen MAPCs were thawed and resuspended in NR8383 media, and 200,000 MAPCs in 0.1 ml or vehicle added to transwells. Plates were cultured for an additional 24 or 48 hours. Transwells and MAPCs were discarded. NR8383 cells in suspension were transferred to 1.5 ml cryovials and centrifuged at 300 × g for 7 minutes. 350 μl of RTL lysis buffer +Beta-mercaptoethanol (Qiagen) was added to plate wells to lyse adherent cells, and to cryovials to resuspend and lyse cell pellets. Lysates were stored at −80 °C. RNA was isolated using Qiagen RNAeasy kit, as described below. 500 ng of RNA was used for cDNA preparation, as described below.

### Time-lapse dish preparation

Delta-T cell culture dishes (Fisher) were prepared as previously described^58^. Culture dishes were rinsed with sterile water and then coated with poly-L-lysine (0.1 mg/ml, Sigma-Aldrich) overnight at room temperature, rinsed with sterile water, and allowed to dry. Aggrecan spot gradients were formed by allowing 2 μl of aggrecan solution (0.7 mg/ml in HBSS-CMF, Sigma-Aldrich) to dry onto the culture surface. The surface of the dish was bathed in laminin solution (10 μg/ml in HBSS-CMF, Invitrogen) for 3 hr at 37°C. The laminin bath was subsequently removed and cells were plated without allowing the surface of the dish to dry.

### Dorsal root ganglion neuron culture

DRGs were harvested as previously described^60^. Briefly, DRGs were dissected from adult female Sprague-Dawley rats (Harlan) and incubated in a solution of collagenase II (200 U/ml, Worthington Biochemical Corporation) and dispase II (2.5 U/ml, Roche Diagnostics) in Ca^2+^/Mg^2+^ free Hank’s Balanced Salt Solution (HBSS-CMF, Invitrogen). Cells were centrifuged at 1000–2000 RPM, washed and gently triturated in HBSS-CMF three times. Dissociated DRGs were then resuspended in Neurobasal-A media supplemented with B-27, Glutamax, and penicillin/streptomycin (Invitrogen). DRGs were plated in Delta-T dishes prepared as described above at a density of 800 cells/cm^2^.

### Macrophage preparation for time-lapse microscopy

NR8383s were cultured as described above, harvested with trypsin/EDTA (Invitrogen), washed three times, and plated in uncoated 6 well plates at a density of 1*10^6^/ml in serum-free F-12 K media or MAPC conditioned media (Athersys) for 24 hours. MAPC conditioned media was created by plating MAPC at 350,000 cells per T175 in serum-free F-12 k media for 48 hours, and media was collected and centrifuged. Before use in time-lapse experiments, the cultured macrophages were harvested with EDTA and a cell scraper, washed three times, and resuspended in Neurobasal-A with HEPES (50 μM; Sigma- Aldrich) at a concentration of 2.5*10^5^/70 μl.

### Time-lapse microscopy

Prior to time-lapse imaging, adult neurons were incubated in 5% CO_2_ at 37 °C for 48 hours in Neurobasal-A media with HEPES (50 μM; Sigma-Aldrich). Before imaging the media was replaced. Imaging was performed at 10 × every 5 minutes using Leica fluorescence stereomicroscopes equipped with heated environmental chambers maintained at 5% CO_2_ at 37 °C. Growth cones were chosen that extended straight into the spot rim and had characteristic dystrophic morphology for at least 60 minutes of baseline. Macrophages, prepared as described above, were then added to the time-lapse dish and observation continued for 17–20 hours.

### Contusive spinal cord injury and animal care

All procedures were approved by the Case Western Reserve University animal resource center and IACUC and carried out in accordance with the approved guidelines. Adult female Sprague-Dawley rats (225–250 g) were obtained from Harlan and acclimated to the animal resource center, behavior analysis chambers, and handlers. Rats were injected intraperitoneally with ketamine (60 mg/kg) and xylazine (10 mg/kg). The musculature was cut form T7-T9 and the dorsal surface of T8 was exposed by laminectomy. The vertebral column was stabilized by clamping the T7 and T9 vertebral bodies with forceps fixed to the base of the Infinite Horizon Impact Device. The animals were situated on the platform, and the 2.5 mm stainless steel impactor tip was positioned over the midpoint of T8 and impacted with 250 kDyne force. The overlying musculature was sutured closed, and the skin was closed using wound clips. The animals were treated with Marcaine at the incision site. The force/displacement graph was used to monitor impact consistency and any animals that exhibited an abnormal impact graph or greater than 10% deviation from 250 kDyne were immediately excluded from the study. Manual bladder expression was performed 2–3 times daily for two-three weeks until a voiding reflex returned and the animals could urinate voluntarily.

### Preparation and injection of MAPCs

MAPCs were obtained from Athersys, Inc. (Cleveland, OH) and stored in the vapor phase of liquid nitrogen. Prior to injection, the MAPCs were thawed, washed, and resuspended in saline. Cells were counted and checked for viability via Trypan blue exclusion. For intraspinal injections, immediately following contusive injury three injections of 2*10^5^ MAPCs were delivered to the lesion center, 1 mm rostral, and 1 mm caudal via a Nanoject II (Drummond Scientific Company) prior to wound closing. For intravenous MAPC delivery, MAPCs were injected into the tail vein either immediately after injury or one-day post injury at the designated dose (1 × 10^5^, 4 × 10^5^, 1 × 10^6^, 4 × 10^6^, or 8 × 10^6^ cells) in 200 μl saline. SCI control animals received saline vehicle injection alone at the same designated time points as the MAPC treated animals.

## Behavioral analysis

### Basso Beattie Bresnahan (BBB) open field locomotor assessment

Two blinded observers conducted all behavior analysis. BBB score and subscore were performed as described previously^38^,^44^. Each animal was tested on days 1, 4, 7 and weekly thereafter through week 10. Animals were allowed to freely roam in an open field while being examined by two expertly trained observers and scored according to the BBB guidelines. Predetermined exclusion criteria stipulated that animals with a BBB score of greater than one at day 1 be removed from the study, but no animals in this study fit these criteria. Data was quantified as the average of the two hind limbs.

### CatWalk

Animals with a BBB ≥10 were included in this analysis. One saline animal was removed. Computer assisted gait analysis (CatWalk, Noldus Information Technology) marked the footprints of 3-4 uninterrupted and constant pace crossings per animal. The regularity index is a quantification of interlimb coordination calculated from the number of normal step sequences and the total number of steps.

### Thermal hyperalgesia

Hyperalgesia analysis was performed at 10 weeks post injury by a blinded observer, as described previously^72^. Animals were given 30 minutes to acclimate to the plexiglass cage prior to testing (Ugo Basile). The IR source (Intensity = 58) was carefully placed under each hind paw. Time to withdrawal was recording as an average of 5 trials on each paw, with the longest and shortest time removed.

### Metabolic cage micturition

At 4, 6, 8, and 10 weeks after SCI, animals were placed over night in a metabolic cage (Braintree Scientific) with access to ample food and water for the measurement of voiding patterns. Urine was collected on a force transducer and strain gauge (Grass Technologies) and plotted in Spike2 (Cambridge Electrical Design, sampled at 20 HZ). Micturition pattern analysis included the frequency of voiding per 16 h and the volume per void. The total volume of expelled urine was not included because of variation of water intake between individual animals.

### Urodynamics

Terminal urodynamic recordings were performed similar to Cheng *et al.* 2004^73^. Briefly, rats were anaesthetized at 10 weeks post SCI with 0.8 g/kg urethane delivered subcutaneously. A polyethylene-50 catheter was carefully inserted through the urethra into the bladder for the delivery of saline. Fine wire electrodes (0.003′′ diameter Teflon-insulated silver wire; A-M Systems) were inserted percutaneously via the vagina on both sides of the urethra to monitor the EUS electromyography (EMG) activity. The electrodes were connected to a preamplifier (HZP; Grass-Technologies), which was connected to an amplifier (QP511, Grass-Technologies) with high- and low-pass frequency filters at 30 Hz and 3 kHz and a recording system (Power 1401, Spike2; Cambridge Electronic Design) at a sample frequency of 10 kHz. Continuous cystometrograms (CMGs) were collected using constant infusion (6 ml/hr) of room temperature saline (Aladdin-1000 single syringe infusion pump; World Precision Instruments) through the catheter into the bladder to elicit repetitive voids. The bladder pressure was recorded via the same catheter used for saline infusion, using a pressure transducer (P11T, Grass Technologies) connected to the recording system at a sample frequency of 2 kHz.

### Perfusion and immunohistochemistry

Rats were transcardially perfused with ice-cold 4% paraformaldehyde in PBS, and the spinal cords and bladders were dissected. The tissue was postfixed in 4% paraformaldehyde overnight at 4 °C and then cryoprotected with 30% sucrose. Spinal cords were then frozen in OCT mounting media and sectioned on a Hacker or Leica cryostat at a thickness of 20 μm. Mounted sections were washed three times with PBS followed by blocking in 5% normal goat serum (NGS) or normal donkey serum (NDS) with or without 0.1% bovine serum albumin (BSA) in PBS. 0.1% Triton-X was added to the blocking buffer depending on the antigen used. Following blocking, sections were incubated in primary antibody diluted in blocking buffer overnight at 4 °C. Primary antibodies used were goat anti-Arginase 1 (1:200, Santa Cruz), mouse anti-ED1 (1:200, Millipore), or mouse anti-GFAP (1:500, Sigma-Aldrich). The next day, the sections were washed with PBS and incubated in the appropriate secondary antibody conjugated to Alexa fluor 488, 594, or 633 (1:500, Molecular Probes) overnight. Sections were then washed, stained with DAPI (1:1500 in PBS, Sigma-Aldrich), washed, and coverslipped. Pixel intensity was measured on images taken on a standard fluorescent microscope (Leica, Germany) with a uniform exposure setting and analyzed using ImageJ.

### Histological analysis

Blinded investigators conducted all tissue staining analysis. Spared white matter analysis was conducted by Eriochrome cyanine (EC) staining as previously published^74^. Briefly, room temperature 20 μm sections were placed in fresh acetone for 10 minutes, removed and allowed to dry for 30 minutes. Sections were stained with freshly filtered EC solution (Sigma-Aldrich) for 30 minutes and washed in running tap water for 5 minutes. The stain was differentiated in 5% ferric ammonium sulfate (Sigma-Aldrich) for 15 minutes and again washed with running tap water for 5 minutes. The differentiation was completed with borax-ferricyanide solution (Sigma-Aldrich) for 10 minutes and briefly washed with running tap water. Slides were dehydrated in 70%, 95%, and 100% ethanol for 3 minutes each followed by xylene for 3 minutes. Slides were coverslipped with VectaMount Permanent Mounting Medium (Vector Laboratories). Sections were digitized with a Leica SCN 400 slide scanner. Twenty-one 20 μm coronal sections (one of every 10 serial sections was stained, 200 μm between sections) both rostral and caudal of lesion epicenter were analyzed for a total of 8 mm of spinal tissue. Sections were traced in ImageJ (NIH) for volume calculation. The section with the least white matter staining was designated as the lesion center for this and all histology at 10 weeks post injury.

Tissue sparing was measured using GFAP. Five 20 μm thick sections, every 1 mm through the lesion, were stained with GFAP and analyzed for intact spinal cord tissue. Images were taken on a standard fluorescent microscope (Leica, Germany). Sections were traced in ImageJ for volume calculation.

GFAP, ED1, and Arg1 were quantified by staining intensity. Five 20 μm thick sections (every 1 mm through the lesion) immunostained with GFAP, ED1, or Arg1, were quantified in ImageJ by subtracting background from the total intensity. We confirmed overlap of Arg1 staining with ED1 staining in the same sections to confirm Arg1 expression was that of macrophages.

### Biodistribution

MAPCs were labeled with the Qtracker® 625 Labeling Kit (Life Technologies). Briefly, frozen MAPCs were thawed in a 37 °C water bath and resuspended in 10 ml MAPC media (Athersys, Inc.) per 5–10 million cells. They were centrifuged 5 minutes at 863 × g at RT. The media was aspirated and the cells were resuspended in media to a concentration of 10 million cells/ml. In a separate tube, 1 μl each of Qtracker A and B were mixed together for each million cells, and incubated at RT for 5 minutes. Then, 200 μl of media per million cells was added to the Qtracker mixture and mixed well. The resuspended cells were added to the Qtracker mixture and incubated at 37 °C for 1 hour with gentle mixing. The cells were then washed twice with 10 m of media each time as above by centrifugation and aspiration and finally resuspended in DPBS (Gibco) at 20 million cells/ml and stored on ice until injection. Six rats were injected with 4 million MAPCs 1 day following injury, as described above. The lungs, liver, spleen, and spinal column were collected 24 or 48 hours following injection, weighed, and frozen in liquid nitrogen until processed by BioInVision using CryoViz technology^55^. An additional rat was injected with 10 million cells 1 day following injury, and harvested 24 hours later for whole rat imaging.

### RNA preparation

Spleens and 5 mm of the spinal cord injury were dissected and frozen on dry ice at sacrifice. Blood was collected in RNAprotect Animal Blood Tubes (Qiagen) at the same time. Tissues and blood were stored at −80 °C until use.

Frozen cords and spleens were weighed, and sufficient RLT Lysis Buffer (Qiagen) with 10 μl/ml 2-Mercaptoethanol (Sigma-Aldrich) was added on ice. A Hard Tissue Disposable Rotor Stator Generator probe (Omni International) homogenized each sample on ice with a final homogenate of <50 mg/ml tissue. The homogenate was treated with the RNEasy Mini kit (Qiagen) according to the manufacturer’s protocol, using the optional DNAse (Qiagen) step. RNA was eluted in 30 μl for spinal cords and 50 μl for spleens. RNA concentration was determined with the Nanodrop and stored at -80°C.

RNA from blood samples was extracted using the RNEasy Protect Animal Blood Kit (Qiagen) according to the manufacturer’s protocol. RNA was eluted into 30 μl and concentration was determined with the Nanodrop. It was stored at −80 °C. 10 μg of RNA from each sample was used with the GlobinClear Mouse/Rat kit (Ambion) according to the manufacturer’s protocol. The RNA was eluted in 30 μl, its concentration determined on the Nanodrop, and stored at -80°C.

Universal rat reference RNA (Agilent) was prepared by centrifuging at 12,000 × g for 15 minutes at 4 °C, removing the supernatant, washing the pellet in 70% ethanol, centrifuging again at 12,000 × g for 15 minutes at 4 °C, removing the supernatant, air drying the pellet for 30 minutes at room temperature, and resuspending in RNAse-free water (Agilent) to 500 ng/μl. It was then stored at −80 °C.

Peripheral blood mononuclear cells (PBMCs) were prepared from rat blood by Ficoll-Paque gradient. Cells were washed with DPBS and resuspended in RPMI, 10% FBS. To further enrich T cells, PBMCs were added to a tissue culture treated dish for 30 minutes at 37 °C to allow non-T cells to adhere. The supernatant was then collected and pelleted, and PBMCs were processed with an RNEasy Mini Kit as described above for frozen cords and spleens, and eluted in 30 μl. RNA concentration was determined with the Nanodrop (Thermo Scientific) and samples were stored at −80 °C.

### Microarray analysis

RNA was prepared as described above and provided to the Gene Expression and Genotyping Facility in the Comprehensive Cancer Center at Case Western Reserve University for array analysis. The RNA was run on the Rat Gene 1.1 ST Array Plate (Affymetrix). R statistical language with Oligo and Limma packages (Bioconductor) were used for RMA normalization and assessment of differential expression between the different conditions. Differential expression was calculated based on moderated t statistics with a Bayesian adjusted denominator. For genes significantly modulated by MAPC treatment (p < 0.05), Qlucore Omics Explorer (Qlucore) was used to generate and analyze the principal component analysis, unsupervised agglomerative hierarchical clustering, and heat map gene expression data. This gene set was then analyzed using Ingenuity Pathway Analysis (Ingenuity) to generate predicted activation state of up stream regulators and cellular functions.

### qPCR

For the RT reactions, 20 μg Random Primers at 500 μg/ml (Promega) were diluted with 1 ml of Nuclease Free Water (Promega) to make a stock solution. 10 μl of stock solution was added to each well in a 96-well microplate (Denville Scientific). 40 μl total of RNA and Nuclease Free Water was added (for 3-day spinal cord samples – 236 ng RNA; for 3 day blood samples –500 ng RNA; 3-day spleen samples – 500 ng RNA; for Universal Rat Reference RNA –500 ng RNA; for Rat PBMCs preparation –125 ng RNA), the plate was covered with an adhesive strip (Qiagen) and put in a 96-well thermocycler at 70 °C for 1 minute then 16 °C for 5 minutes. While at 16 °C the program was paused and 30 μl of RT mix, consisting of 14 μl of MMLV RT 5X Buffer (Promega), 3.5 μl of dNTP mix (made by mixing 150 μl each of 100 mM dATP, dTTP, dGTP, and dCTP (Promega) with 900 μl of Nuclease Free Water (Promega)), 0.7 μl 40 U/ml RNAsin (Promega), 2 μl of 200 U/μl MMLV RT (Promega), and 9.8 μl of Nuclease Free Water (Promega), was added to each well. The plate was covered with another adhesive strip and the thermocycler resumed to complete the cycle: 16 °C for 5 minutes, 37 °C for 15 minutes, 42 °C for 15 minutes, 45 °C for 15 minutes, 92 °C for 2 minutes, and then a hold at 4 °C. While held at 4 °C, 1 μl of RNAse-IT (Agilent) added to 9 μl of TE buffer was added to each well. The temperature was then ramped to 37 °C for 30 minutes and then cooled to 4 °C. The cDNA plates were stored at −20 °C until use. –RT control reactions were created in the same manner but with the 200 U/μl MMLV RT replaced with Nuclease Free Water (Promega).

qPCR was run using the GoTaq qPCR Master mix kit (Promega) in 96-well PCR microplates (Axygen). cDNA from the RT reactions was diluted 5-fold in Nuclease Free Water (Promega). Primers (Table 2) were resuspended in Nuclease Free Water (Promega). Spinal cord was tested for expression of Ly6c, MMP7, and NOS2. Blood was tested for NOS2, CTLA4, CXCL10, and FoxP3. Spleen was tested for NOS2, CTLA4, LAG3, ISG54, and ISG60. GAPDH was used as a reference gene. Universal Rat Reference was used for normalization with all targets, except for FoxP3, which was normalized to Rat Blood PBMCs. NR8383s were tested for expression of NOS2 and Arg1, and normalized to beta-actin expression. All samples were run in triplicate.

Each well contained 12.5 μl SYBR Green (Promega), 7.2 μl Nuclease Free Water (Promega), 0.4 μl CXR (Promega), 5 μl of diluted cDNA, and 0.15 μl each of forward and reverse primers (Integrated DNA Technologies). The plate was covered with MicroAmp Optical Adhesive Film (Applied Biosystems) and run on a 7500 Fast Real-Time PCR System (Applied Biosystems) using the 7500 Fast System Software (Applied Biosystems) with the following program: Step 1–50 °C 2 minutes, Step 2–95 °C 10 minutes, Step 3–95 °C 15 seconds, Step 4–60 °C 1 minute, Step 5–Repeat steps 3–4 40 times. A dissociation was added to the end: 95 °C for 15 seconds, 60 °C for 1 minute, 95 °C 15 seconds. The data were initially analyzed with the software and then exported to Excel (Microsoft) compatible format for further workup. The data were further analyzed by taking the average CTs for the detector in question and subtracting the average CTs for GAPDH of the same sample to give dCT. The Universal Rat Reference for the detector had the same function performed. The Universal Rat Reference dCT was then subtracted from the sample dCT to give ddCT. The formula 2^^(−ddCT)^ was used to determine the relative expression for each animal. For FoxP3, Rat Blood PBMC was used instead of the Universal Rat Reference.

### Data analysis

All statistical analyses were performed using Prism (Graphpad) and considered significant when p < 0.05, unless otherwise noted. All results are presented as mean ± s.e.m. D’Agostino–Pearson and Shapiro–Wilk tests were first performed to determine normality of data. For BBB score, BBB subscore, and metabolic cage analysis a two-way repeated measures ANOVA was performed to compare all injured groups. Post-hoc analysis between Saline and treated groups at individual time points were performed with Fisher’s Least Significant Difference post-hoc test. The Catwalk regularity index was performed on animals with a BBB ≥10 (1 saline removed). D’Agostino–Pearson and Shapiro–Wilk tests found all three groups were non-normally distributed. ROUT analysis identified one animal as an outlier, and was removed from analysis (MAPC 0 dpi, CatWalk regularity index = 29.63). A Kruskal-Wallis ANOVA was performed with Dunn’s post-hoc test comparing Saline and treated groups. Urodynamic data was evaluated via ANOVA between injured groups with Fisher’s Least Significant Difference post-hoc test. Histological quantification was analyzed using two-tailed Student’s t-test at each distance from lesion epicenter comparing saline to MAPC treated. Microarray analysis used R statistical language with Oligo and Limma packages (Bioconductor) for RMA normalization and assessment of differential expression between the different conditions. Differential expression was calculated based on moderated t statistics with a Bayesian adjusted denominator. Ingenuity Pathway Analysis was used to calculate a statistical z-score for the predicted activation state of up stream regulators. The bias-corrected z-score was used for z-scores flagged as bias. Further details of the z-score calculations can be obtained at http://pages.ingenuity.com/rs/ingenuity/images/0812%20upstream_regulator_analysis_whitepaper.pdf. Ingenuity Pathway Analysis employed a right-tailed Fisher Exact Test to calculate the p-value of cellular functions modulated by MAPC treatment. qPCR data was analyzed in all groups via two-way ANOVA with Fisher’s Least Significant Difference post-hoc test. Spleen weight data was analyzed by two-tailed Student’s t-test between injured groups. *In vitro* macrophage gene expression was analyzed by ANOVA comparing all culture conditions with Fisher’s Least Significant Difference post-hoc test. Spot dieback assay was analyzed by two-tailed Student’s t-test.

## Additional Information

**How to cite this article**: DePaul, M. A. *et al.* Intravenous multipotent adult progenitor cell treatment decreases inflammation leading to functional recovery following spinal cord injury. *Sci. Rep.* **5**, 16795; doi: 10.1038/srep16795 (2015).

## Supplementary Material

Supplementary Information

Supplementary Movie 1

Supplementary File 1

Supplementary File 2

## Figures and Tables

**Table 1:** Distribution of animal groups.

Route	Timing	Treatment	Total#	#removed	# completedstudy
intraparenchymal	0 dpi	Saline	9	0	9
intraparenchymal	0 dpi	6 × 10^5^ MAPC	12	0	12
intravenous	0 dpi	Sham	22	1	21
intravenous	0 dpi	Saline	20	3	17
intravenous	0 dpi	MAPC	10	1	9
intravenous	1 dpi	4 × 10^6^ MAPC	19	3	16
intravenous	1 dpi	1 × 10^5^ MAPC	7	0	7
intravenous	1 dpi	4 × 10^5^ MAPC	8	0	8
intravenous	1 dpi	1 × 10^6^ MAPC	9	0	9
intravenous	1 dpi	8 × 10^6^ MAPC	8	0	8
		Failed surgery/injection	35		

All animals received a 250 kdyne impact at thoracic level 8. MAPC was administered either immediately following injury (0 dpi), or one day post injury (1 dpi) at the specified dose and route. Animals were immediately excluded from the study if surgical complications arose, such as failed intravenous injections or abnormal contusion hits as defined by the force/displacement graph of the Infinite Horizon Impactor (n = 35). Animals were excluded from the study if they developed bladder infections or other health complications (n = 8). All animals that reached the predetermined endpoints are present in this study.

**Table 2:** qPCR primers used in this study.

	Forward	Reverse
GAPDH	GATGCTGGTGCTGAGTATGT	CCACCCTTCAGGTGAGC
Ly6c	TGCGCTATGAAGTCCTGTATG	GTGGGACTTCTATGCAACTGTA
MMP7	CCACTGAACTTCAAGAGGGTTA	GTTTCCTGGCCCATCAAATG
NOS2 (*in vivo*)	CACATCTGGCAGGATGAGAAG	CGCATTAGCACAGAAGCAAAG
CTLA4	CCCAATCTTCTCTGAAGCCATAC	ACCTCATCAGTGTTGTGTGAAG
CXCL10	CATTCCTGCAAGTCTATCCTGT	GCTCTTGATGGCCTCAGATT
ISG54	AGTTGAGTGTGCTTCCAGAG	AGACTGCTCTCCAATGATTCC
ISG60	CAGCCTATTCCTGACATCCTATG	CACTTCACAGCACATCTCTCTC
LAG3	AGTGTACACCGCAGAGTCTA	AGGATGAGAGAGAGGAAGAGATG
FoxP3	AGCACCTTTCCAGAGTTCTTC	GAGGGTGGCATAGGTGAAAG
Beta Actin	AGGCCCCTCTGAACCCTAAG	CAACACAGCCTGGATGGGCTAC
Arg1	CAGCAGGAACCCTGGATGA	AAAGGCGCTCCCATAATCTCT
NOS2 (*in vitro*)	CATCAGGTCGGCCATTACTGT	CCAGATCCGGAAGTCATGCT
