# Supplementary material for: Intravenous multipotent adult progenitor cell treatment decreases inflammation leading to functional recovery following spinal cord injury
Source: Sci Rep. 2015 Nov 19;5:16795. doi: 10.1038/srep16795 (PMC4652166; doi:10.1038/srep16795)
Supplement: Supplementary Information [file srep16795-s1.pdf]

Supplementary Data

Intravenous multipotent adult progenitor cell treatment decreases inflammation leading to functional recovery following spinal cord injury

DePaul, Marc A.[1]; Palmer, Marc[2]; Lang, Bradley T.[1,2]; Cutrone, Rochelle[2]; Tran, Amanda P.[1]; Madalena, Kathryn M.[1]; Bogaerts, Annelies[3]; Hamilton, Jason A.[2]; Deans, Robert J.[2]; Mays, Robert W.[2]; Busch, Sarah A.[2], Silver, Jerry[1*]

[1]Case Western Reserve Univ., Dept. of Neurosciences, 10900 Euclid Ave., SOM E654, Cleveland, OH, 44106, USA

[2]Athersys, Inc. Regenerative Medicine, Cleveland, OH, 44115, USA

[3]ReGenesys, Bioincubator Leuven, 3001, Leuven, Belgium

*Corresponding author jxs10@case.edu

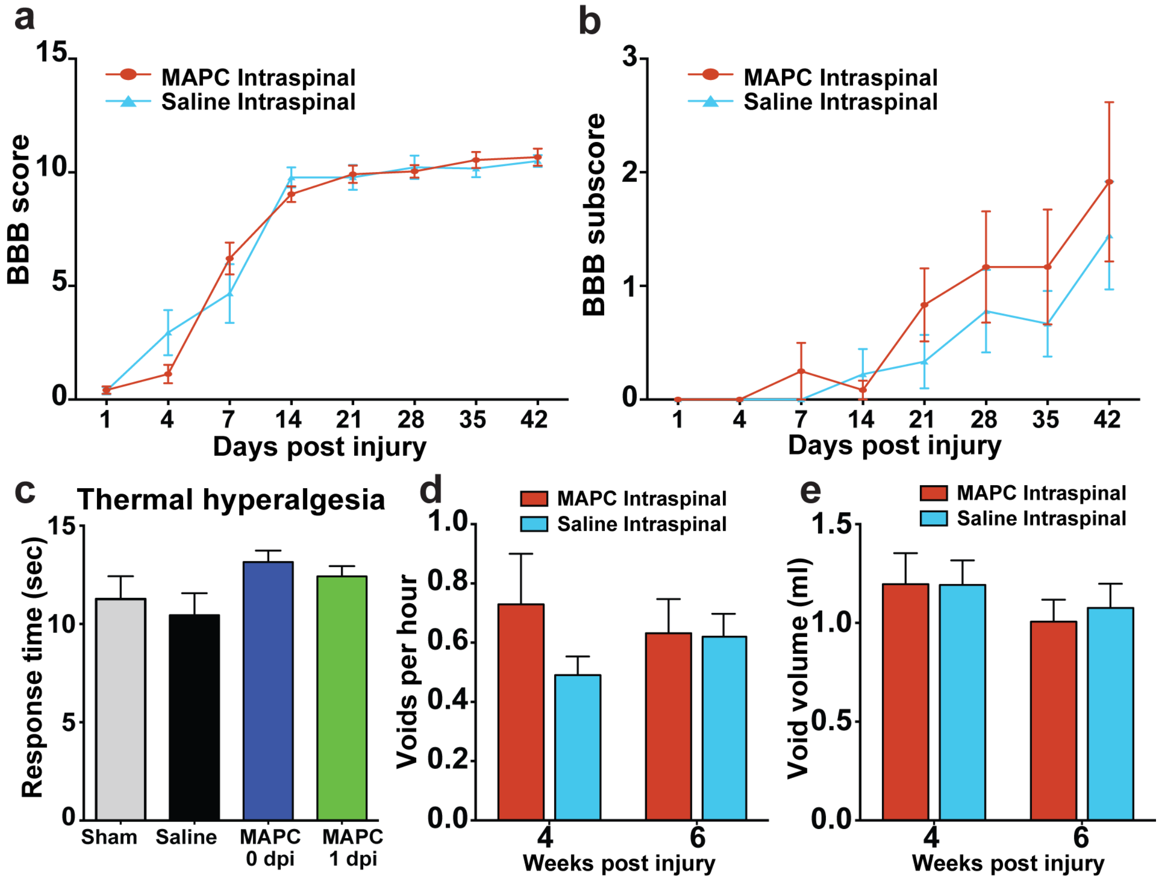

Supplementary Figure 1. Intraspinal MAPC treatment did not improve physiological recovery following SCI. Intravenous MAPC treatment of contusive spinal cord injury did not induce hyperalgesia. (A-B) Locomotor recovery following intraspinal injections as measured by the BBB scale and BBB subscale. *n* = 9 saline, 12 MAPC. Two-way repeated measures ANOVA. (C) Average response time to withdraw paw from thermal stimuli (Hargreave's test) of intravenous treated animals at 10 weeks after injury. *n* = 6 Sham, 7 Saline, 9 MAPC 0 dpi, 8 MAPC 1 dpi. One-way ANOVA. Data represent mean and s.e.m. (D-E) Metabolic cage quantification of average void frequency and void volume of intraspinal treated animals. *n* = 9 Saline, 12 MAPC. Two-way repeated measures ANOVA.

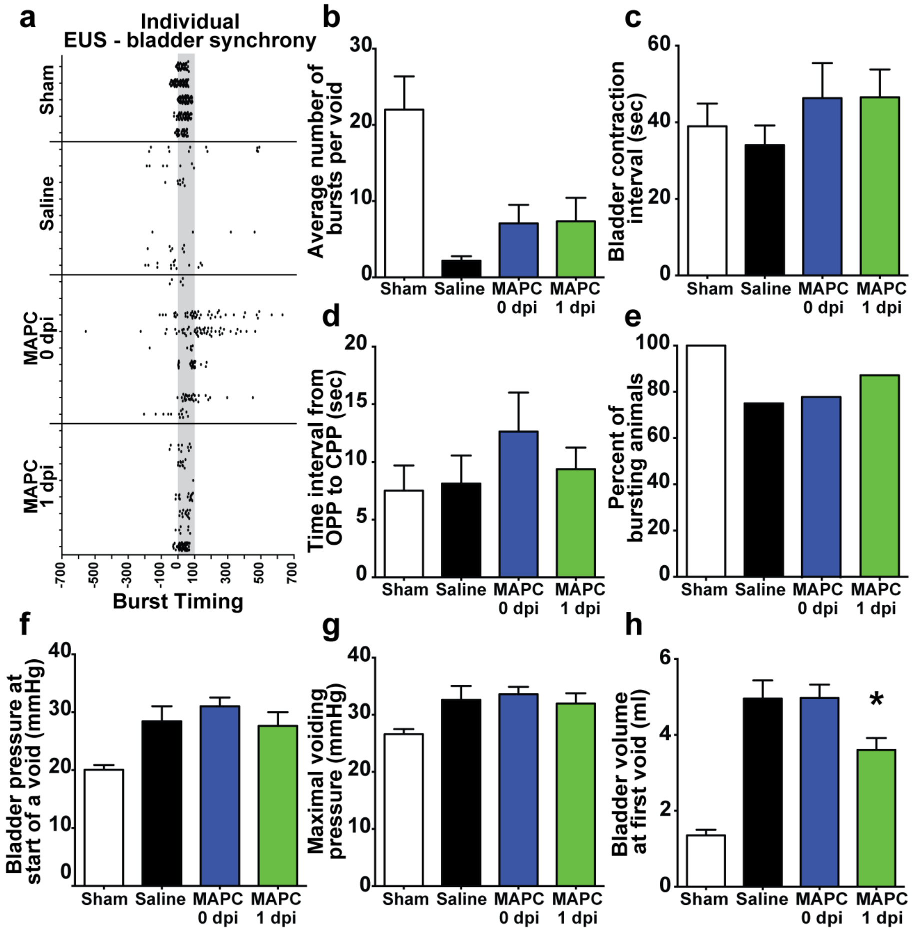

Supplementary Figure 2. MAPCs improve bladder-EUS coordination and urodynamics after SCI. (A) Individual representation of EUS bursting over three void cycles in relation to bladder contractions. Grey box denotes the time interval between OPP and CPP (0%-100%) (B) Average number of EUS bursts per void. (C) Length of time from the start to the end of a bladder contraction. (D) Length of time between OPP and CPP. (E) Percent of animals displaying EUS bursting. (F) Pressure at which a void was initiated. (G) The maximal pressure reached during a void. (H) The bladder volume needed to initiate a void. *n* = 5-6 Sham, 8 Saline, 9 MAPC 0 dpi, 8 MAPC 1 dpi. *p<0.05, ***p<0.001 when compared to Saline treated. One-way ANOVA, Fisher's Least Significant Difference post-hoc test. Data represent mean and s.e.m.

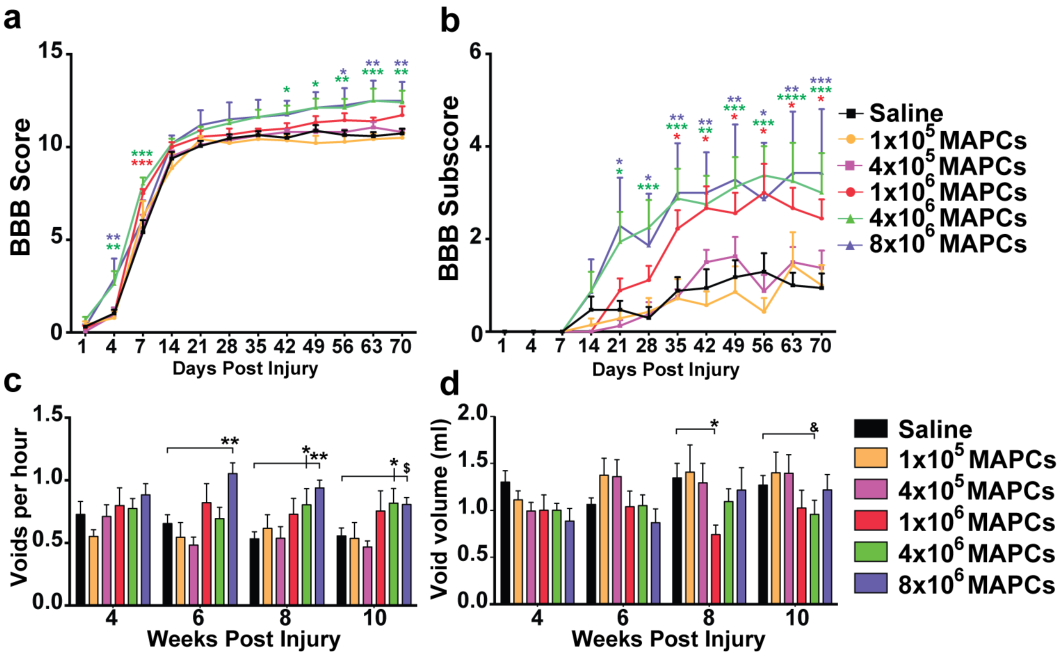

Supplementary Figure 3. MAPCs improve physiological recovery in a dose dependent manner. The (A) BBB scale and (B) BBB subscale of functional locomotor recovery of saline or increasing intravenous MAPC doses delivered one day following SCI. *n* = 17 Saline, 7 1x10^5 MAPC, 8 4x10^5 MAPC, 9 1x10^6 MAPC, 16 4x10^6 MAPC, 8 8x10^6 MAPC. *p<0.05, **p<0.01, ***p<0.001, ****p<0.0001 when compared to Saline treated. Two-way repeated measures ANOVA, Fisher's Least Significant Difference post-hoc test. (C-D) Metabolic cage quantification of void frequency and void volume of saline or increasing intravenous MAPC doses delivered one day following SCI. *n* = 17 Saline, 7 1x10^5 MAPC, 8 4x10^5 MAPC, 9 1x10^6 MAPC, 16 4x10^6 MAPC, 8 8x10^6 MAPC. $^{\$}$p=0.089, $^{\&}$p=0.071, *p<0.05, **p<0.01 when compared to Saline treated. Two-way repeated measures ANOVA, Fisher's Least Significant Difference post-hoc test. Data represent mean and s.e.m.

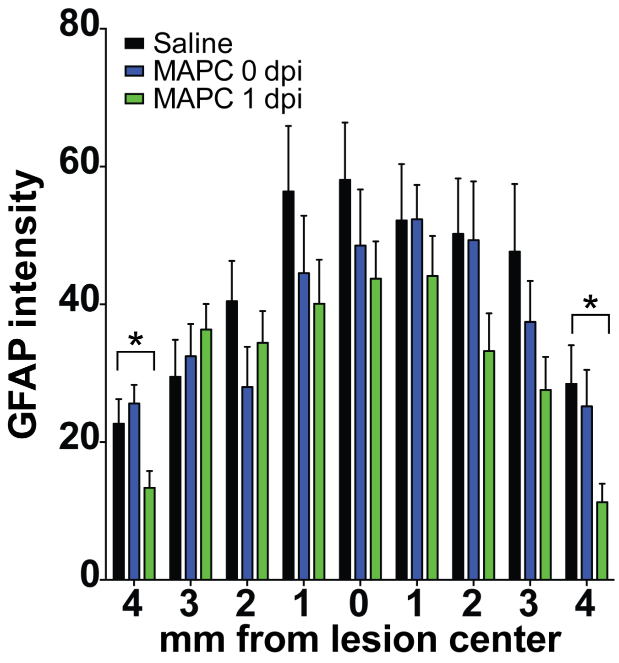

Supplementary Figure 4. MAPCs reduce astroglial reactivity distal form the lesion epicenter. Quantification of astroglial reactivity ten weeks post injury as measured by GFAP staining intensity. *n* = 8 Saline, 9 MAPC 0 dpi, 8 MAPC 1 dpi. *p<0.05 when compared to Saline treated, Student's t-test. Data represent mean and s.e.m.

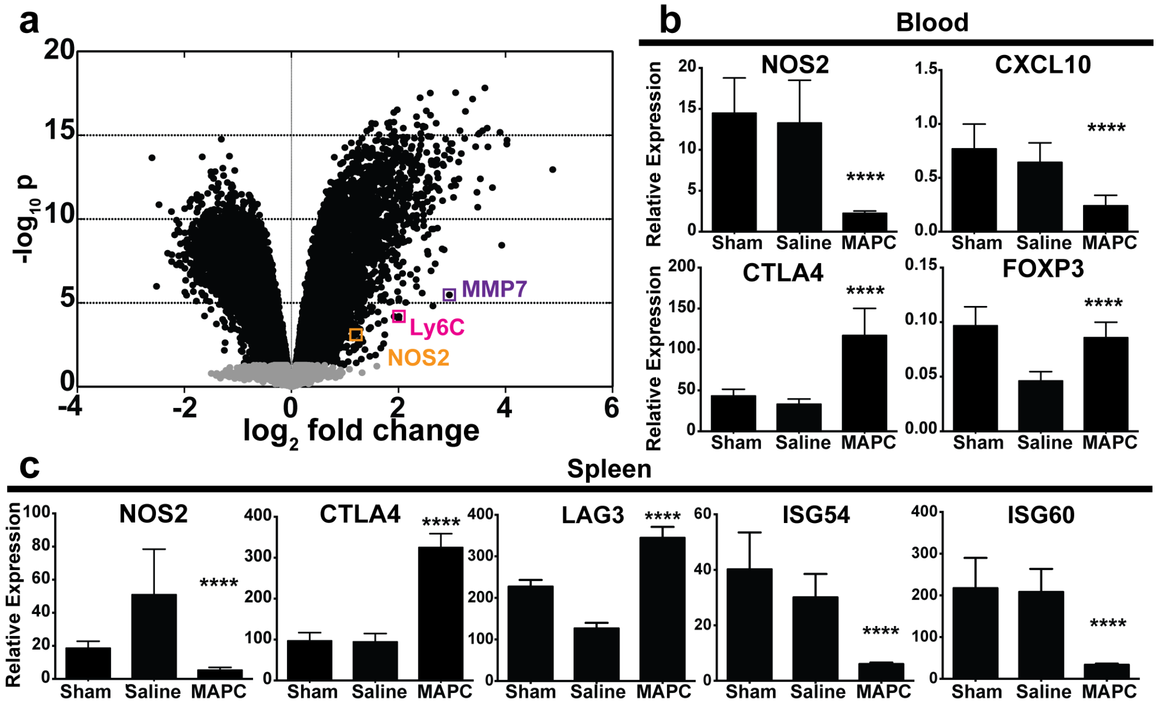

Supplementary Figure 5. Spinal cord microarray analysis and qPCR of peripheral systems. (A) Volcano plot showing differentially expressed genes at the injury site following injury plus saline treatment compared to sham. Black circles $p<0.05$, grey circles $p>0.05$, t-statistic with Bayesian adjusted denominator. (B-C) Expression of select genes four days post SCI following Saline or MAPC 1 dpi treatment, as determined by qPCR and normalized to GAPDH in (B) the blood, and (C) the spleen. $n = 6$ per group, 3 biological replicates per animal. ****$p<0.0001$ when compared to Saline treatment. Two-way ANOVA, Fisher's Least Significant Difference post-hoc test.

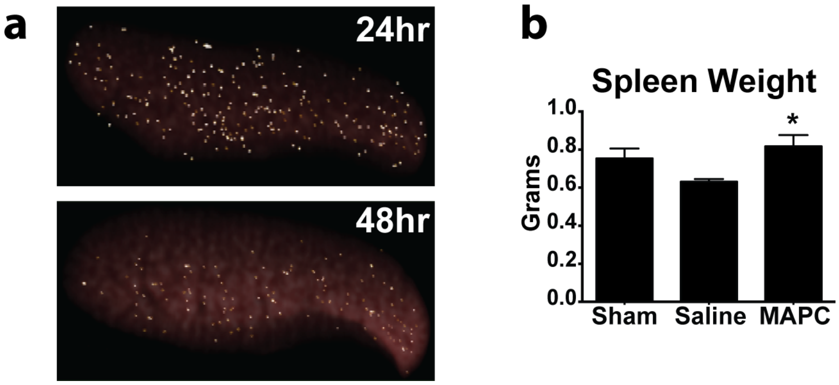

Supplementary Figure 6. MAPCs home to the spleen and preserve splenic mass. (A) Detection of Qdot labeled MAPCs decreases rapidly from 24 to 48 hours post injection. 3D representative fluorescent CryoViz images of Qdot labeled MAPCs in the spleen 24 and 48 hours post intravenous cell administration. (B) Spleen weight four days post injury. *n* = 6 per group. *p<0.05 Student's t-test. Data represent mean and s.e.m.

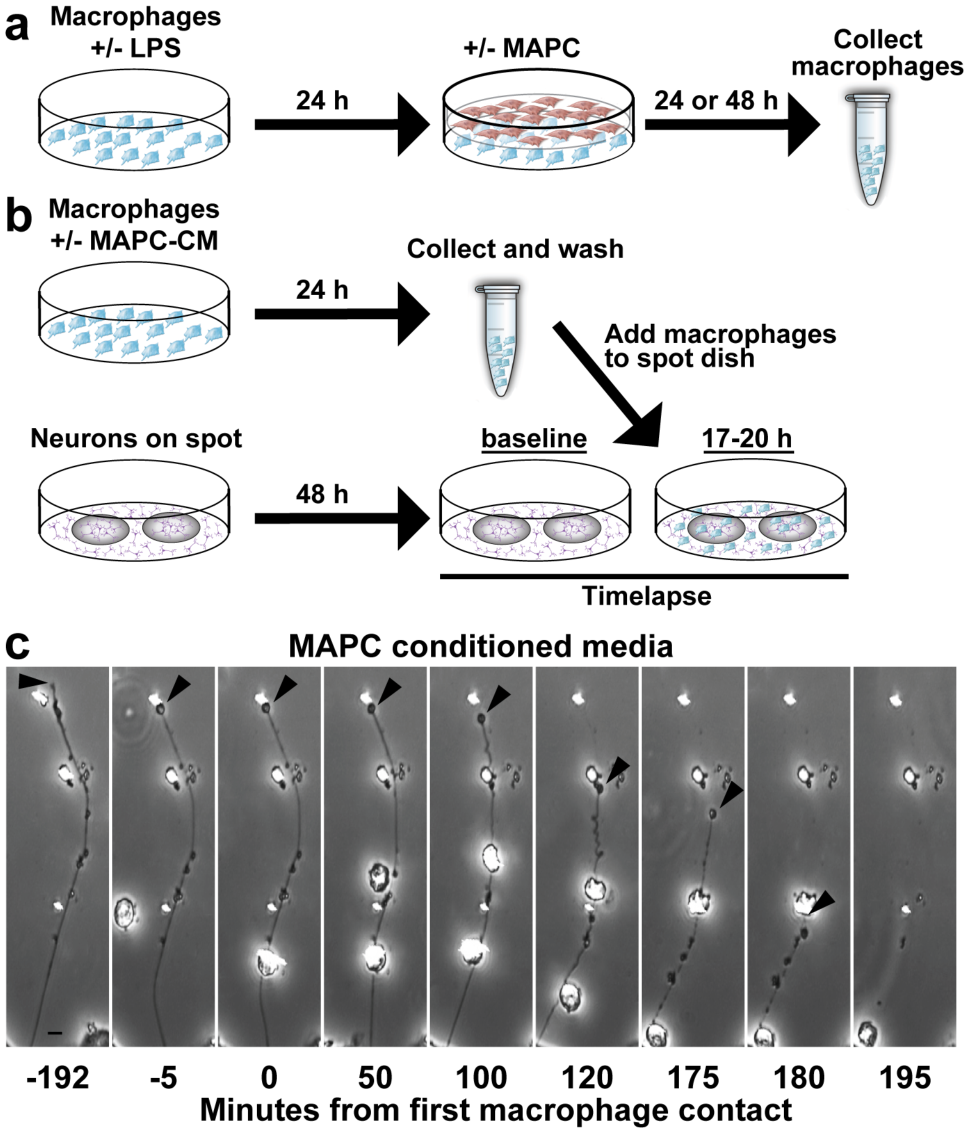

Supplementary Figure 7. *In vitro* experimental design and delayed dieback after MAPC-CM treatment. (A) *In vitro* gene expression experimental design. NR8383 macrophages were cultured with or without LPS for 24 hours. MAPC or vehicle was then added in transwell and cultured for 24 or 48 additional hours followed by macrophage collection and analysis by qPCR. (B) Dieback assay experimental design. Dorsal root ganglion neurons were plated on chondroitin sulfate proteoglycan gradient spots on laminin and cultured for 48 hours prior to time-lapse observation. Neurons were observed at baseline followed by addition of macrophages and 17-20 hours of additional

observation. Macrophages were cultured in MAPC conditioned media or control media for 24 hours, collected and washed prior to addition to neuronal culture. (C) Representative time-lapse of delayed macrophage mediated axonal dieback from MAPC-CM pretreated macs. Arrowhead denotes tip of axon. Scale bar = 10μm.

Supplementary Movie 1. Movie of the spine and surrounding tissue 24 hours post Qdot labeled MAPC intravenous injection 1 dpi. Video begins in the cervical spinal cord and moves caudal to the lumbar cord, before returning to the cervical cord. Note, no cells are found in the spinal cord.

Supplementary Data 1. Normalized expression values from the microarray analysis for genes significantly modulated by MAPC treatment compared to saline.

Supplementary Data 2. Ingenuity Pathway Analysis results of significantly modulated upstream pathways and cellular functions in MAPC treated animals compared to saline.
